# Data publication: towards a database of everything

**DOI:** 10.1186/1756-0500-2-113

**Published:** 2009-06-24

**Authors:** Vincent S Smith

**Affiliations:** 1 Natural History Museum, Cromwell Road, London, SW7 5BD, UK

## Abstract

The fabric of science is changing, driven by a revolution in digital technologies that facilitate the acquisition and communication of massive amounts of data. This is changing the nature of collaboration and expanding opportunities to participate in science. If digital technologies are the engine of this revolution, digital data are its fuel. But for many scientific disciplines, this fuel is in short supply. The publication of primary data is not a universal or mandatory part of science, and despite policies and proclamations to the contrary, calls to make data publicly available have largely gone unheeded. In this short essay I consider why, and explore some of the challenges that lie ahead, as we work toward a database of everything.

## Discussion

Journal articles as immutable, citable, archives of knowledge, have been, and continue to be, the mainstay of scholarly communication. Viewed by many scientists as the end product of their engagement in a piece of research, the "article" contains an argument or statement about an hypothesis, backed up by supporting data. However, as new technologies drive research toward larger and more complex datasets, these two features of the journal article are becoming increasingly disarticulated [1]. In some scientific disciplines – for example crystallography, astronomy and molecular biology – digital repositories have become important avenues for "publishing" data. This approach has found common cause with social and political forces that are arguing for greater accountability and transparency of science. The Open Science movement for the free use (and re-use) of data, results and protocols, is championed by many as the best way to improve the collective societal return on our investment in scientific research [2]. Data publication is widely recognised as being central to delivering this. But in truth, outside a handful of disciplines, publication of science data is the exception, not the rule.

Data publication has the potential to deliver significant benefits from local to global scales. Organisations and research disciplines can benefit from increased recognition [3]. There are significant potential cost savings for funders through greater reuse of data [4], and economic benefits by stimulating entrepreneurial uses of data by commercial companies [5]. Data publication can help to discourage scientific misconduct [6], and in many cases (e.g. environmental and ecological data) provides the only outlet for data that are irreplaceable because of the unique circumstances in which they were collected. So why, when so much is to be gained from data publication, do scientists compromise scientific development, and effectively leave their work unfinished by not publishing their data? I argue that it is not through lack of money or policy that scientists behave in this way. Likewise, misunderstandings and inertia with the scientific community are only partly to blame. A more likely cause is that the benefits to an individual of making their data publicly available are less evident to the scientist than they are to the governments, funding agencies and scientific community that support them. Only by addressing this imbalance, and making these benefits immediate and transparent to practising scientists, will data publication become the norm. Here are three suggestions on how this can be achieved:

### Make it easy – developing the cyberinfrastructure

For those scientists for whom data publication is possible, it is too often considered a chore. Of course it is dangerous to generalize across a multiplicity of scientific disciplines, each with their own specialised norms and practices, but as a taxonomist and systematist generating molecular, morphological and phylogenetic data in support of my biodiversity research program, my experience with data publication systems has always been a painful affair. Almost without exception they require a substantial time investment, sometimes involving personal contact with a remote database manager who massages my data into a form such that it can be readily parsed. If data publication is to become a part of normal scientific practice it has to be easy to achieve. This requires a robust infrastructure that is quick and simple to use, works with the applications and data formats currently employed, and gives the scientist confidence that it will work and still be there when needed. Data standards are part of this process, but perhaps more important is the development of robust applications that hide the complexity of these data standards through a well designed interface. Funding agencies need to respond to these infrastructural needs, which have to be maintained beyond the typical lifecycle of a standard grant application if they are to have lasting impact. Related to this is a need for a career path and recognition structure for those informaticians who develop the software and standards associated with these systems [7]. Without this human infrastructure, the data, computational and communication components of this cyberinfrastructure cannot be sustained.

### Make it citable – motivating data publication through peer recognition

A primary motivation for article publication is to demonstrate the authors' contribution to science [8]. This attracts peer recognition that influences the authors' reputation, employment and research opportunities. Article citation is the most common metric of peer recognition and if a comparable metric could be brought to bear on data publication, it follows that value and impact of data publication could be similarly tracked to motivate authors.

At present data publication where possible, is largely motivated by enforcement through the editorial practices of particular journals. These require that authors lodge data in a suitable repository as a prerequisite to publication. In this instance the citation of data usually takes the form of an opaque identifier (e.g. the GenBank accession number [9] or Web site URL) rather than the data authors or editors in a manner equivalent to a traditional article citation. This failure to cite the authors of an original data source has plenty of precedent: for example, publications that describe new species are rarely mentioned in subsequent studies. If they were, the scientific contributions of taxonomists would be amongst the most cited articles worldwide. Opaque identifiers will continue to be required for data publication for practical reasons, since large datasets are increasingly collaborative, often involving many hundreds of authors. Nevertheless, data publishers should be able to demonstrate the same editorial standards as article publishers, by making the authors' and editors' names and addresses readily accessible, preferably in a way that can be read by both humans and machines for computation of citation metrics. Not only would this introduce greater transparency and accountability in science, but through peer recognition, motivate authors to publish their data.

### Make it useful – moving beyond data archival

One reason for publishing data is to archive those data in a form that it is available to others for reuse. This activity however, has little value for the contributor who already holds the data and may have to exert considerable effort to publish it. Automatically enhancing the value of the data to the contributor once it has been published can address this problem. This may be in form of functional enhancements that facilitate the subsequent manipulation, editing and annotation of the data, or semantic enrichments that automatically connect data to other published sources [10]. This fusion of data might take the form of descriptive metadata that assists in data discovery, connection to definitions of concepts and terms found within the data set, and enhanced visualisations of data. Importantly, these enhancements should be reciprocal between linked data, enriching the value of old and new data alike as the knowledgebase grows. Not only does this enhance the discoverability of published data but, because these links are machine readable, it can facilitate the computation and (perhaps eventually) the semantic reasoning across the links.

How can we achieve all this with a multiplicity of distributed stakeholders, many of whom have conflicting or competing interests? To my mind, data stewardship is best accomplished in systems and repositories where the custodian has trusted status within relevant communities of practice. Such trust is earned with difficulty and lost with ease; therefore it makes most sense to place these repositories with scientific societies, institutions and journals that have a history of supporting, archiving and enabling these communities. This is counter to the trend toward large national data centres that must accommodate the diverse interests of potential contributors spanning many broad scientific disciplines. Scientific data exist in many types and formats, and is subject to varying legal, cultural, protection and practical constraints. They are often used in different ways according to their context and have varying life-cycle requirements. Who better to understand these needs than the communities that are generating and using the data. This, however, risks the construction of data silos – walled parochial gardens of disciplinary data that remain unconnected to the wider world.

## Conclusion

The power of published data is amplified by ingenuity through applications and uses unimagined by the original authors and distant from the original field. Without connecting these disparate datasets the true potential of data reuse and repurposing is lost. New data integration services are already emerging, transforming data discovery on the web from lists of search results into tools that compute answers to structured questions. Recent high profile examples include Google Squared [11] and Wolfram Alpha [12]. These offer a vision of what a database of everything might look like, drawing on public data amassed from parochial datasets, scientific journals, encyclopaedias, repositories and other sources freely available on the Web. At present these tools have the predictability and efficiency of a database, but lack many of the fundamental features relevant to data publication. There is no easy way of publishing to these tools, no means to correct errors, little attribution of sources, and no means of data citation. Crucially the results returned from tools like Google Squared and Wolfram Alpha lack the social and intellectual context necessary to judge the value of the data. Until these deficiencies can be addressed our database of everything, with all its shortcomings, is likely to look similar to the way it does now – scholarly articles, published in scholarly journals, by researchers too busy and unmotivated to publish all but a minimum of their underlying data.

## About the Author

Vince Smith is a cybertaxonomist at the Natural History Museum, London and is part of the EU funded European Distributed Institute of Taxonomy (EDIT). He created and manages the Scratchpad project , a data publication framework and social networking tool supporting research communities of biodiversity scientists.
